# Supplementary material for: A Look Under the Carpet of a Successful Eradication Campaign Against Small Ruminant Lentiviruses
Source: Pathogens. 2025 Jul 20;14(7):719. doi: 10.3390/pathogens14070719 (PMC12299675; doi:10.3390/pathogens14070719)
Supplement: Supplementary file 1 [file pathogens-14-00719-s001.zip › pathogens-3744096-supplementary.pdf]

**Questionnaire for Determining the Status of SRLV A in Goats**

**General Farm Information:**

- TVD Number of the Farm: _________ (if multiple locations, please provide additional TVD numbers: ___________________)
- Are you a member of SZZV?
  - Yes
  - No
- Farm Type: (* Multiple answers possible)
  - Milk
  - "Hobby"
  - Other: ______________
- Current Number of Goats: _________
  - Female: _____
  - Male: _____
- Number of Goats in 2016: _________ (approximately)
- Number of Goats in 2011: _________ (approximately)

**Breeding:**

- Do you have your own offspring?
  - Yes
  - No

**Purchasing Practices:**

- Do you buy foreign goats?
  - Yes
  - No (If no, proceed to question 11)
- How many animals do you buy on average per year?
  - Bucks: _____
  - Goats: _____
  - Kids: _____
- Where do the purchased animals come from? (* Multiple answers possible)
  - Private purchase
  - Public market/show
  - Other: _____________
- From which country do the purchased animals come? (* Multiple answers possible)
  - Switzerland
  - Abroad (please specify the country of origin): ______________
- Were the animals tested for CAE (SRLV) before purchase?
  - Yes, all
  - Yes, some of them
  - No
  - Unknown

**Contact with Other Animals:**

- Do you send your goats to summer pastures?
    - Yes
    - No (If no, proceed to question 15)
- Do goats have contact with animals from other herds during this period? (* Multiple answers possible)
    - Yes, with foreign goats
    - Yes, with foreign sheep
    - No
- Are there periods outside of summer pastures when your goats graze with foreign herds?
    - Yes
    - No (If no, proceed to question 15)
- Which animals do the goats have contact with during this period? (* Multiple answers possible)
    - Foreign goats
    - Foreign sheep

**Sheep Co-habitation:**

- Do you keep sheep on your farm?
    - Yes
    - No (If no, proceed to question 21)
- If yes, number of sheep: ________
- Production form of sheep farming: (* Multiple answers possible)
    - Milk
    - Meat
    - Other: ___________________
- Do you keep the goats together with the sheep on your farm at times?
    - Yes
    - No (If no, proceed to question 20)
- When do the animals have contact with each other? (* Multiple answers possible)
    - Shared stable
    - Shared pasture
    - Other contact (please describe): ______________________________
- Can newborn goat kids steal milk from sheep (cross-suckling)?
    - Yes
    - No
- Have your sheep been tested for Maedi Visna (MV)?
    - Yes
    - No
- If yes, have you ever had MV-positive sheep on your farm since 2010?
    - Yes
    - No

**MVV-positive Goats:**

- In the past, goats on your farm have tested positive for SRLV A (Maedi-Visna Virus), e.g., in the full survey of 2011/2012 or during random sample tests in subsequent years.
- Handling of MVV-positive goats:
    - What was done with the MVV-positive goats immediately after the positive test result?
        - Eliminated
        - Kept in the herd (possibly no longer in the herd)
    - If kept in the herd (* Multiple answers possible):
        - Still in the herd
        - Offspring still in the herd
    - Were the offspring also tested positive for MVV?
        - Yes
        - No
        - Not tested
        - No offspring
    - If yes, ID of the offspring: ____________________
    - If multiple goats were MVV-positive, please describe on the back what was done with each goat if the procedure was not uniform.

**Thank you for your cooperation!**

Let me know if you need any further assistance! 😊
